# Knowledge and Acceptance of Human Papillomavirus Vaccine for Cervical Cancer Prevention Among Urban Professional Women in Bangladesh: A Mixed Method Study

**DOI:** 10.1089/biores.2018.0007

**Published:** 2018-05-01

**Authors:** Afsana Bhuiyan, Farhana Sultana, Jessica Yasmine Islam, Mohiuddin Ahsanul Kabir Chowdhury, Quamrun Nahar

**Affiliations:** ^1^ London Northwest Healthcare Trust, Harrow, United Kingdom.; ^2^ Victorian Cytology Services Registries, East Melbourne, Australia.; ^3^ Centre for Epidemiology and Biostatistics, Melbourne School of Population and Global Health, University of Melbourne, Carlton, Australia.; ^4^ Department of Epidemiology, Gillings School of Global Public Health, University of North Carolina at Chapel Hill, Chapel Hill, North Carolina.; ^5^ International Centre for Diarrheal Disease Research, Bangladesh (icddr, b), Dhaka, Bangladesh.

**Keywords:** Bangladesh, cervical cancer, HPV, human papillomavirus, HPV vaccine, knowledge

## Abstract

Prophylactic human papillomavirus (HPV) vaccination is the most effective preventive method against invasive cervical cancer, the second leading cause of cancer-related deaths among women in Bangladesh. Evidence on women's knowledge and perception about cervical cancer and HPV vaccination are needed for effective implementation of national cervical cancer prevention programs. The objective of this study was to assess the knowledge, attitude, and acceptance of cervical cancer, HPV, and HPV vaccination among urban professional women in Bangladesh. We recruited 160 female professionals employed at selected private banks in Bangladesh. Participants were selected using nonprobability-based convenience sampling for interviews through a self-administered questionnaire. Later, in-depth interviews were conducted with nine of these women. Quantitative data were analyzed utilizing descriptive statistics, whereas qualitative data were analyzed using a thematic approach. Ninety-eight percent of participants reported that they had previously heard of cervical cancer, however, only half (51%) reported to have heard of HPV as a cause of the disease. Less than 1% of the 160 participants had previously undergone a pap smear, and only 2% were vaccinated with at least one dose of HPV vaccination. Although knowledge was low, intention for acceptance of vaccination was moderate for women and high for their children. Although the majority of women had heard of cervical cancer, few women had in-depth knowledge of HPV and the etiology of invasive disease. This study draws attention to the urgent need of educational interventions on cervical cancer and its prevention to improve uptake of available HPV vaccination in Bangladesh.

## Background

Globally, each year an estimated 500,000 women are diagnosed with cervical cancer, resulting in over 270,000 deaths.^1^ Although highly preventable, cervical cancer occurs most frequently in low-resource countries, where almost 9 out of 10 cervical cancer cases (87%) occur.^1^ In South Asia, the age-standardized incidence rate of cervical cancer is 18.9 per 100,000 women, compared to an average of ∼10 per 100,000 in the developed world.^2,3^ Cervical cancer is the second most common cancer among Bangladeshi women with 12,000 new cases annually.^4^ Similar to other low-resource settings, the burden of cervical cancer in Bangladesh is grossly underestimated due to the unavailability of national cancer registries and undiagnosed cases, which are known to be very common in Bangladesh.^5^ Limited hospital data from Bangladesh show that about one-quarter of cancer-associated deaths among women are due to cervical cancer, which is not reflected in national estimates.^6,7^

Persistent infection with high-risk human papillomavirus (HPV) has been established as a necessary cause of cervical cancer.^8^ Globally, 70% of cervical cancer cases are due to high-risk subtypes HPV-16 and 18.^9^ Infection with another six common high-risk subtypes 31, 33, 35, 45, 52, and 58 in various combinations with 16 and 18 are responsible for 90% of the disease burden worldwide.^10^ Primary prevention of cervical cancer by HPV vaccination and secondary prevention by screening have been proved to be the two most effective ways to prevent invasive cervical cancer.^11^ In most developed countries, organized screening has led to a reduction in incidence of cervical cancer with early detection and successful treatment of precancerous cervical lesions.^12,13^ However, regular screening is beyond the scope of most developing countries due to high costs, limited health infrastructure, competing health policy priorities and limited healthcare personnel.^5,14–16^ HPV vaccination, on the contrary, provides an opportunity to low-resource settings, such as Bangladesh, to reduce the burden of cervical cancer through primary prevention and successful HPV vaccination uptake among the target population of adolescents.^12^

In 2016, HPV vaccination was introduced for the first time in Bangladesh by the Ministry of Health, with the support from the Global Alliance for Vaccines and Immunizations (GAVI). Vaccination introduction program will run for 2 years in one district of the country, and if it is successful, GAVI will provide support for national introduction of HPV vaccination.^17^ In order to establish a successful national HPV vaccination strategy, data are needed on women's knowledge and attitudes toward cervical cancer and its prevention, across different segments of society. In early 2018, data using a cross-sectional study of Bangladeshi women with low educational attainment (mean: ∼7 years of education) and low income showed, despite poor knowledge of cervical cancer, willingness to obtain HPV vaccination was very high.^18^ One other previous report of Bangladeshi women has documented high (81%) awareness of cervical cancer, however, the majority (74%) of these data were collected from women residing in rural areas and limited to women above the age of 30 years; however, this study found high awareness, but did not explore willingness to vaccinate oneself or their daughters.^19^ On the contrary, in another African study,^20^ low awareness was related to increased willingness to vaccinate one's child, but not oneself. To our knowledge, no previous studies have been conducted to assess the knowledge of cervical cancer and HPV, and acceptance of HPV vaccination with a focus on Bangladeshi women with high educational background and high socioeconomic status. Data from this particular subgroup are needed to tailor educational programs for optimal uptake of HPV vaccination in Bangladesh. In this study, our primary aim was to assess knowledge, source of information, and acceptance of HPV vaccination. As a secondary aim, we assessed the barriers and facilitators in acceptance of vaccination in a group of highly educated professional women in urban Dhaka.

## Methods

**FIG. 1.**
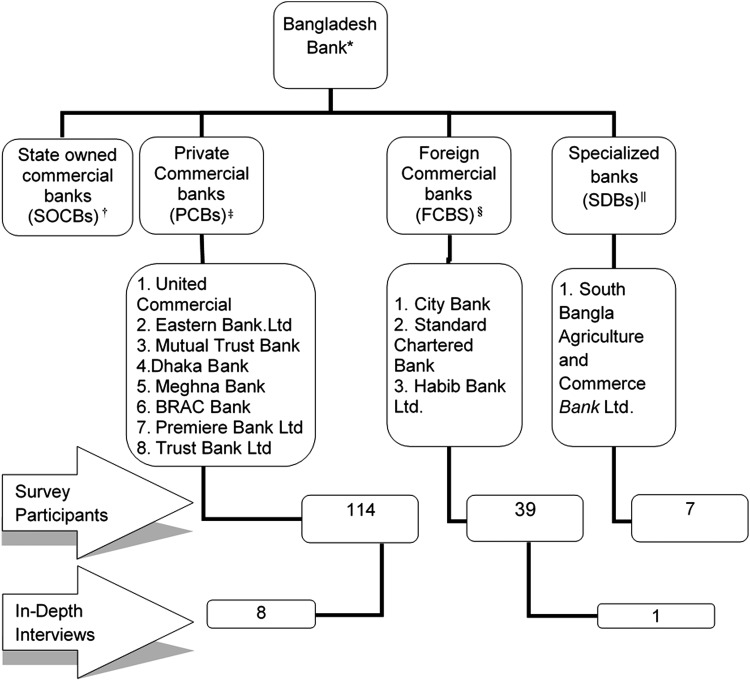
Summary of banks targeted for recruitment and the flow of enrollment. *Bangladesh Bank is the central bank of Bangladesh and the chief regulatory authority in the nation's banking sector. Bangladesh Bank oversees the functioning of all other banks in the country, which fall under four major branches. ^†^SOCBs are fully or majorly owned by the Government of Bangladesh. ^‡^PCBs are majorly owned by private entities. They perform the banking functions in conventional manner that is, interest-based operations. ^§^FCBs are banks which are incorporated abroad with permission to function in Bangladesh. ^||^SDBs are banks established for specific objectives such as agricultural or industrial development. These banks are fully or majorly owned by the Government of Bangladesh. BRAC, Bangladesh Rural Advancement Committee.

We conducted a mixed-methods study in December 2013 in the capital city of Bangladesh, Dhaka. The study included two components: (1) an initial structured questionnaire, followed by (2) in-depth interviews (IDI). Professional women employed by private banks in Dhaka (above the rank of cashier) were recruited using nonprobability based convenience sampling and recruited through mail, email, phone, or in-person. Figure 1 provides a summary of banks targeted for recruitment and the flow of enrollment.

A self-administered structured questionnaire was used, which included a total of 35 questions. The questionnaire was adopted from a previously administered survey of a population-based sample of female adults in Bangladesh.^18,21^ Sociodemographic information such as age, marital and educational status, religion, average monthly income, and whether they had a daughter were collected. The survey included questions to assess knowledge of cervical cancer and HPV and knowledge of and attitude toward HPV vaccination.

Initially, women were asked, “Have you ever heard of a cancer called cervical cancer?” If a participant answered yes, then a series of questions followed to assess knowledge on cause and prevention of the disease. If they responded “no”, indicating that they had never heard of cervical cancer, then questions on knowledge of cause and prevention of cervical cancer were skipped. Next, participants were asked if they had ever heard of a virus named HPV. If participants responded yes, then a series of questions to assess detailed knowledge of the etiology of HPV and its relationship with cervical cancer were asked. However, if they responded “no”, then these questions were skipped as well. Finally, participants were asked if they have heard of a vaccine to protect against HPV and if they answered “Yes,” they were further assessed; otherwise the interview was stopped. Specific questions were also asked to assess willingness toward receiving HPV vaccination. In addition, women were asked for suggestions on how awareness can be improved (Table 1).

**Table 1.** **Component Interview Questions of Structured Questionnaire**

Knowledge questions	Potential responses
Have you ever heard about cervical cancer?	Yes/no
From where did you hear about cervical cancer?	Open ended
Did anyone in your family/friends suffer from cervical cancer?	Yes/no
Do you know the cause of cervical cancer?	Yes/no If yes, then open ended
Do you know the measures to prevent cervical cancer?	Yes/no If yes, then open ended
Have you heard about a virus named human papillomavirus or HPV?	Yes/no
Do you know how a person can get HPV infection?	Handshake, kissing, shared objects, blood, sexual contact/open ended
What are the disease(s) that can be caused by HPV?	Hand warts, genital warts, oral cancer, cervical cancer, respiratory infection/open ended
Have you heard about the HPV vaccine?	Yes/no
Acceptance	
Are you interested to get HPV vaccine?	Yes/no If no, why not—open ended
Do you think even after getting HPV vaccine you will still have chances to develop cervical cancer?	Yes/no
Do you think there are any side effects of vaccine?	Yes/no If yes, specify—open ended
Suggestion and practice	
Would you recommend this vaccine to others?	Yes/no
Would you like to vaccinate your daughter(s) to have it?	Yes/no
Do you think more women need to be aware about cervical cancer?	Yes/no
Do you think more women need to be aware about HPV vaccine?	Yes/no
What are the ways to increase awareness about cervical cancer and HPV vaccine?	Open ended

HPV, human papillomavirus.

Face-to-face IDIs were conducted with selected participants of the survey. Women were purposefully selected based on their survey responses to obtain a representative sample based on the following demographic variables: marital status, HPV vaccination status, and whether or not the woman had a daughter. This method allowed for a discussion on a range of issues applicable to women of varied demographics, including knowledge and awareness of HPV and cervical cancer, including women's preference for the vaccination (if they have not had one), and provided the interviewer with opportunities to probe deeper into certain topics of interest, prompted by the participant. For example, if a participant reported that a family member(s) was affected by cervical cancer, that woman was further questioned to assess the impact of the disease on the family and her decision to obtain HPV vaccination.

An interview guide was developed by the research team to assess the following topics: (1) knowledge of cervical cancer and its causes; (2) knowledge of HPV and its transmission; (3) knowledge on HPV vaccine and source of information; (4) acceptance of HPV vaccine; and (5) barriers and facilitators of acceptance of HPV vaccination. Initially, all participants were asked the following questions: 1. “Would you please tell me what you know about cervical cancer?”; 2. “Have you heard about a virus named human papillomavirus or HPV?”; 3. “Do you know the relationship between human papillomavirus or HPV and cervical cancer?”; 4. “Have you ever been vaccinated with HPV vaccination?” Subsequently, open-ended questions were asked to explore specific issues and women were probed on each topic till saturation was reached. All interviews were conducted by the first author (A.B.). The IDIs were conducted in Bangla. The interviews were tape-recorded, and later transcribed and translated into English by A.B. and Q.N., who are fluent in both English and Bangla.

### Data analysis

Univariate descriptive analyses were completed to summarize the survey data. Frequencies and percentages were reported to describe data gathered on knowledge and attitudes, including distribution of the sample by different demographic characteristics. The survey data were entered into Microsoft Excel 2012 and analyzed using STATA version 12.

Qualitative data analysis was completed in different stages, adopting Braun and Clarke's thematic analysis approach: data familiarization, coding, looking for themes, reviewing themes, and producing a final report.^22^ A list of *a priori* codes were initially developed based on the interview guidelines and later modified after reviewing a few interviews. These codes were used to code all the transcripts. The coding was done manually and independently by two researchers (A.B. and Q.N.) and later assessed for intercoder reliability. The data were then arranged in clusters as per codes and grouped as per themes and subthemes.

## Results

From a total of 12 private banks located in Dhaka, 180 women were approached and 160 of them agreed to participate (89%) (Fig. 1). The basic demographic characteristics of these women are described in Table 2. The mean age of study participants was 31 years (SD ±6.35 years). The majority (73%) were ever married, and more than three-quarters had a postgraduate degree or higher. About 60% of participants had a monthly income of BD taka 30,000 or more (1 USD = 78 BD taka), and about a quarter of them had a daughter. Sixteen women reported of someone in their family being affected by cervical cancer (Data not shown).

**Table 2.** **Background Characteristics of Survey Respondents (*N* = 160)**

Characteristic	*n*	%
Age of participants (years)		
<28	61	38
29–39	84	52.5
>40	15	9.4
Mean age with SD: 31 ± 6.35		
Marital status		
Ever married	116	72.5
Unmarried	44	27.5
Religion		
Muslim	151	94.4
Hindu	7	4.4
Christian	2	1.2
Educational status		
Bachelors or equivalent	35	21.9
Masters or higher	125	78.1
Monthly income		
10,000–30,000	59	36.9
30,001–50,000	45	28.1
>50,000	45	28.1
Have a daughter	36	22.5

SD, standard deviation.

Out of the nine participants chosen for the IDI, only two were vaccinated and both of these vaccinated women were unmarried. Of those not vaccinated (*n* = 7), one woman had a daughter (unvaccinated), and another woman was 7 months pregnant at the time of the interview. The remaining five were married and had no children.

### Knowledge of cervical cancer and its causes

Ninety-eight percent of participants reported to have heard of cervical cancer (*n* = 157). Participants reported to have heard of cervical cancer from newspapers or magazines (52%), family or friends (36%), and the television (29%) (Table 3). Less than one-fifth reported to have heard of cervical cancer from their doctor.

**Table 3.** **Knowledge of Cervical Cancer**

	*n*	%
Heard of cervical cancer^a^		
Source of knowledge of cervical cancer^b^		
Newspaper or magazine	81	51.6
Family or friends	56	35.7
Television	46	29.3
Doctor	29	18.5
Internet	25	15.9
Radio	10	6.4
Other	2	1.3
Don't know	6	3.8
Know measures to prevent cervical cancer	89	55.6
Measures to prevent cervical cancer^b^		
Condom	14	8.8
Regular checkup	48	30
Pap smear	11	6.9
HPV vaccine	47	29.4

^a^ Total *n* = 157.

^b^ Multiple responses possible; totals may add up to more than 100%.

During the IDI, the concept of a virus causing cervical cancer came as a surprise for most women. These women also mentioned that this was the first time that they heard of HPV.

*“I assumed from this questionnaire that there might be some relationship between the two, but to be honest I know nothing about how a woman gets this disease (cervical cancer).”—*(30-year-old, married, not vaccinated)

### Knowledge of HPV and its transmission

Almost half (*n* = 76) reported that they have heard of HPV. Among those who had heard of HPV, about half were able to identify HPV, a sexually transmitted infection (Data not shown). Among the women who heard of HPV, 67% reported cervical cancer to be a disease caused by HPV, when listed as an option in the structured survey. Very few (*n* = 13) mentioned oral cancer or other cancers related to HPV.

### Knowledge about prevention of HPV infection and cervical cancer

Eighty-nine (56%) participants reported measures to protect themselves against cervical cancer (Table 3). The commonly reported measures to prevent cervical cancer were regular checkup (30%) and the use of HPV vaccination (29%). Only 3% correctly chose all four options (i.e., regular checkup, condom use, regular Pap smears, and HPV vaccine).

Knowledge on the prevention of cervical cancer seems very poor. Some women blamed themselves for being unaware of ways to prevent cervical cancer and some said no one counseled them adequately about the disease. Women reported that their physicians and other healthcare providers they encountered were reluctant to counsel them for screening for cervical cancer.

*“I don't really remember the name of the test but all I know it was very painful and uncomfortable. My doctor advised me to do it every 6 months but I never bothered doing it after I was tested negative the first time. She did not tell anything why to do this or any details of the test. This is the problem with doctors here in Bangladesh. There is no counselling.”*—(35-year-old, unmarried, first assistant vice president, not-vaccinated but had a pap smear)

### Knowledge on HPV vaccination and source of information

When asked about HPV vaccination, 56% of participants reported to have ever heard of HPV vaccination (Table 4). Among those who knew of HPV vaccination, the source of knowledge of HPV vaccination was newspaper or magazine (40%), television (30%), their doctor (29%), and their family and friends (24%) (Table 4).

**Table 4.** **Knowledge and Attitude of Human Papillomavirus Vaccine**

	*n*	%
Heard of HPV vaccine	90	56.6
Source of knowledge of HPV vaccine		
Doctor	26	28.9
Family or friends	22	24.4
Newspaper or magazine	36	40
Television	27	30
Internet	13	14.4
Radio	3	3.3
Other	1	1.1
Don't know	6	6.7
Know number of doses of HPV vaccine	19	21.6
Number of doses (*n* = 19)		
1	2	10.5
3	15	78.9
Other	2	10.5
Know recommended age of HPV vaccine	31	34.8
Recommended age (*n* = 31)		
9–16 Years	5	15.2
Above 16 years	26	78.9
Other	2	6.1
Interested in HPV vaccine	68	43.3
Reason for not previously receiving the HPV vaccine		
Don't know enough	71	78.4
Doctors did not recommend	12	13.6
Worried about safety	4	6.8
Too old for vaccine	5	5.7
Do not need a vaccine	3	3.4
Not sexually active	2	3.4
Too expensive	2	2.3
Recommend HPV vaccine to others	113	70.6
Recommend HPV vaccine to daughter (*n* = 36)	25	69.4
Barriers and facilitators to administering the vaccine		
Chance to develop cervical cancer even after vaccine	11	7
Fear of side effects of the HPV vaccine	3	2

Out of the women who had heard of HPV vaccination, 22% reported they knew the number of doses required for vaccination. However, of those 22%, 79% (*n* = 15) were able to correctly report three doses of vaccination (which is the current schedule in Bangladesh). Only five women could mention the correct recommended age schedule for vaccination of 9–13 years.

A 25-year-old unmarried vaccinated woman admitted her ignorance regarding the ideal age:

“They have told us that this age is the best to receive the vaccine. And if you are older, you have to undergo many other examination procedures. Since most are unmarried, it's best to receive it now, because once married there are more hassles to get the vaccine.”

### Acceptance of HPV vaccination

Forty-three percent of study participants showed interest in receiving HPV vaccination in the future (Table 4). For example, during the IDI, one woman expressed her interest in HPV vaccination:

*“Yes I should take the vaccine and I will recommend others to get it too. If a vaccine can prevent a cancer, we should not think twice about it.”—(*30-year-old, married senior officer, not-vaccinated)

When survey respondents who had previously heard of HPV vaccination (*n* = 90) were asked about reasons for not taking vaccination, the majority said they did not know enough about vaccination (78%), some mentioned that their doctor did not recommend it to them (14%), while a few reported reasons such as worries related to the safety of vaccination (7%) and the fact that they were too old for it (6%) (Table 4). Of those who had one or more daughters, more than half (69%) wanted their daughters to be vaccinated. However, during the IDI, mothers expressed interest in receiving more information regarding the efficacy and safety of the HPV vaccination before making a final decision. One woman noted during the IDI:

*“Such survey itself is I guess a way to increase awareness. Letting us realize that we don't know is also a way to know.”—*(30-year-old, unmarried priority manager, not-vaccinated)

During the IDIs, one of the participants said that most women do not have access to the kind of information they need to make an informed decision on how to prevent the disease. A 30-year-old unmarried woman expressed her interest to know more regarding this issue:

*“Realizing that we don't know is also a way to know. Now I am curious to know about it and would google cervical cancer now and see what it is all about and how this can be prevented.”—(*30-year-old, unmarried priority manager, not-vaccinated)

### Barriers and facilitators in acceptance of vaccination

Very few (2%) participants who have not been vaccinated (*n* = 150) expressed the fear of developing side effects after vaccination; however, none could mention any possible side effects. One woman who was vaccinated believed that her infertility was related to receiving the vaccination as revealed in the IDI.

*“I developed some menstrual problems with abdominal discomfort right after taking the first dose and then when I went for taking the next I asked questions about the side effects. Though I have completed the 3 doses I was having some confusions on the safety. But later I had difficulty in getting pregnant and got diagnosed of endometriosis. My gynecologist told me that it is not as a result of vaccination. I tried googling about my disease and also cervical cancer. I gathered adequate knowledge and found no relation between HPV vaccine and endometriosis but I still think vaccination can be a cause for my infertility.”*—(25-year-old, married, junior officer, vaccinated)

The decision to be vaccinated appeared to be influenced by personal experience, such as a family member previously diagnosed with cervical cancer. One participant was heavily influenced by her mother's diagnosis of cervical cancer. The high cost of vaccination (15,000 BDT) did not prevent her from receiving the vaccine, as she understood its potential benefits.

Ten percent of the participants had a family member or friend who had previously suffered from cervical cancer. Participants who participated in IDI described that they fear cancer. Cervical cancer was described as devastating to the whole family, both emotionally and financially. One participant perceived that the suffering from cancer and its effect on the family depend on the age at which the disease occurs:

*“I had an aunt who suffered from it, and then she was diagnosed at the end stage where there was nothing left to do… What happens is that the whole family suffers… Since she was old, her suffering was a bit less but if a woman of my age suffers from such a disease. Then the family suffering will be horrifying. I have small children….”—*(35-year-old, married, manager, not-vaccinated)

### Suggestions to promote awareness of cervical cancer

Almost all women (99%) reported that there should be programs to raise awareness about HPV and cervical cancer (Data not shown). More than half of women thought reaching out to women through mass media such as television commercials, and house-to-house visit or mass campaigns by healthcare workers would be most effective.

Through the IDIs, women also emphasized the importance of advertisement through mass media, such as television. Some suggested using billboards for advertisement of HPV vaccination, as many people spend considerable time traveling in Dhaka city due to immense traffic.^23^ Other suggestions included seminars or brochures on the topic to disseminate information to women similar to themselves or to other working groups (e.g., garment workers). One woman suggested that men should also be involved in such efforts. Another suggested educational programming through posters in beauty parlors, as women of all age groups and socioeconomic statuses visit them:

*“Why don't you keep posters in parlors? All women go to parlors. There are all kinds of parlors in Dhaka.”*—(35-year-old, married having a 5-year-old daughter, manager, not-vaccinated)

## Discussion

In this study of professional women of high socioeconomic background in Dhaka, Bangladesh, we were able to assess knowledge of cervical cancer and HPV, and awareness and willingness to receive the HPV vaccination. We found that the majority of women had heard of cervical cancer. However, only half of participants had heard of HPV, and even fewer women knew that HPV was sexually transmitted. Only a third of women reported that getting HPV vaccination could prevent cervical cancer, and fewer than half were willing to receive vaccination in the future. However, the majority of women with daughters were willing to get their adolescent daughters vaccinated. Although more than half of participants were not willing to receive vaccination in the future, very few expressed concerns over safety of the vaccination. Women expressed interest in learning more about HPV vaccination to make an informed decision about whether to vaccinate themselves and their daughters. Participants suggested using mass media such as television, healthcare workers, posters, and bill boards as avenues for educational campaigns to reach the target population.

The results of this study suggest that despite high level of education, knowledge of the association of HPV and cervical cancer and relevant preventative methods is low. A systematic review conducted on women's knowledge and attitudes towards HPV vaccination found that factors that affect women's acceptance of HPV vaccination included knowledge of efficacy of the vaccine.^24^ Many women lack knowledge about HPV and cervical cancer, which subsequently impacts uptake of HPV vaccination.^24^ In our study, we found that willingness to have the HPV vaccine in future was moderate for women themselves; however, was relatively high for their daughters. We found that women included in our study expressed interest in receiving more information on vaccination to make an informed decision. Previously published literature has shown similar results.^20,25^ Similar studies conducted in low- and middle-income countries (LMICs) in Africa and South America also showed that despite the difference in sociocultural and other differences, similar results were found in these countries. The majority of participants were unfamiliar with HPV and cervical cancer, but were concerned about their child's and their own risk for HPV and cervical cancer and were willing to vaccinate their child. High rates of vaccine uptake have been previously shown to be associated with high knowledge of HPV, HPV vaccination, and cervical cancer in Australia and the United States. It is therefore crucial that information is made available to women for decision-making.^26^

Results from this study provide insight into the different strategies that can be used to reach out to women among the target population for HPV vaccination and also, catch-up vaccination among older women and mothers of adolescents in Bangladesh. Most women in our study suggested utilizing mass media, seminars, and billboards as means to spread information about the benefits of HPV vaccination. In addition, this study underscores the importance of involving men in educational efforts, as it was mentioned by women during IDI. Previous studies conducted in Bangladesh identified the importance of neighbors and relatives as a source of knowledge of cervical cancer, indicating that social network is an important source of knowledge.^18^ Involving men in educational efforts would improve the social acceptance of discussing HPV and cervical cancer, which may be vital to efforts in the future to vaccinate young boys in Bangladesh. Such efforts have been previously reported to be an effective and important public health strategy for HPV vaccination program success and effective uptake of vaccination among both adolescent girls and boys.^27^

The importance of mass media as a source of information on HPV and its vaccine has also been reported in other studies done in Italy, Belgium, and Malaysia.^28–30^ A study of 575 parents of teenagers assessed the effect of an educational intervention through mass media and revealed that after receiving an educational intervention the percentage of parents in favor of vaccinating their children rose from 55% to 75%.^31^ Similar intervention studies to improve knowledge of cervical cancer and HPV and uptake of vaccination should be conducted in Bangladesh to optimize educational campaigns, as findings from developed countries and other sociocultural background may not be applicable. Newspaper and television appear to be popular media outlets to reach women in Bangladesh. The use of leaflets and newspapers, although effective in highly literate social groups, may be of limited value in populations of lower socioeconomic status. Indeed, a previous study conducted in Bangladesh among women with low education and income found that less than 5% of women, out of almost 2000, reported their source of knowledge of cervical cancer was from newspapers.^18^ Nongovernmental and other charitable organizations should make significant contributions to successfully implement programs to build awareness of HPV vaccination through workshops targeted at relevant segments of the population.

Data from this study also showed that healthcare providers were not an important source of information of cervical cancer, HPV, and HPV vaccination. Similar findings have been reported in other LMIC^32^ and in another study conducted in Bangladesh.^18^ This is of particular significance as evidence suggests that healthcare professional plays a key role in vaccine uptake.^33^ Researchers from the United States have identified that healthcare provider's recommendation of HPV vaccination is a strong predictor of uptake of vaccination.^34^ Educational programs targeted at healthcare providers to inform them of the benefits, efficacy, and costs associated with HPV vaccination should be prioritized.

Most participants were confident that vaccination can prevent disease; however, a few believed that they were still at risk of developing cervical cancer even after vaccination. This is possible given that cervical cancer can be caused by high-risk HPV DNA subtypes other than those included in the vaccine, although a small percentage. Similar findings were revealed in formative research results in Uganda, Peru, Vietnam, and India before HPV vaccination^35,36^ and underscores the necessity of educational campaigns to advise women to continue regular screening.^12^

Several limitations should be taken into consideration when interpreting the results of this study. We utilized nonprobability-based convenience sampling to recruit participants and thus the findings may not be representative to other professional or women with similar education or economic profile. Furthermore, the data collected on history of HPV vaccination and pap smears were based on self-report, which may be prone to social desirability bias. However, this study was the first to be conducted in Bangladesh and provides information on knowledge of HPV and cervical cancer and willingness to take HPV vaccine for themselves and their daughters among a group of highly educated women, working in the banking sector. In addition, we were able to conduct IDI to collect women's detailed views and experiences regarding cervical cancer and HPV vaccination.

## Conclusions

Level of knowledge and awareness of the study participants regarding cervical cancer and HPV vaccination was found to be poor, despite our study population's high educational and socioeconomic status. This reflects the importance of immediate implementation of educational campaigns across Bangladesh before vaccination is made available through a national HPV vaccination program to improve uptake of vaccination and its effectiveness.

## Abbreviations Used

GAVI: Global Alliance for Vaccines and Immunizations
HPV: human papillomavirus
IDI: in-depth interviews
LMICs: low- and middle-income countries
SD: standard deviation
